# A Novel Three-Dimensional Computational Method to Assess Rod Contour Deformation and to Map Bony Fusion in a Lumbopelvic Reconstruction After En-Bloc Sacrectomy

**DOI:** 10.3389/fsurg.2021.698179

**Published:** 2022-01-05

**Authors:** Peter Endre Eltes, Mate Turbucz, Jennifer Fayad, Ferenc Bereczki, György Szőke, Tamás Terebessy, Damien Lacroix, Peter Pal Varga, Aron Lazary

**Affiliations:** ^1^In Silico Biomechanics Laboratory, National Center for Spinal Disorders, Buda Health Center, Budapest, Hungary; ^2^Department of Spine Surgery, Semmelweis University, Budapest, Hungary; ^3^School of PhD Studies, Semmelweis University, Budapest, Hungary; ^4^Department of Industrial Engineering, Alma Mater Studiorum, Universita di Bologna, Bologna, Italy; ^5^Department of Orthopaedics, Semmelweis University, Budapest, Hungary; ^6^INSIGNEO Institute for In Silico Medicine, Department of Mechanical Engineering, The University of Sheffield, Sheffield, United Kingdom; ^7^National Center for Spinal Disorders, Buda Health Center, Budapest, Hungary

**Keywords:** computational method, sacrectomy, bony fusion, bone mineral density, lumbopelvic reconstruction, implant deformation, biomechanics, computed tomography

## Abstract

**Introduction:** En-bloc resection of a primary malignant sacral tumor with wide oncological margins impacts the biomechanics of the spinopelvic complex, deteriorating postoperative function. The closed-loop technique (CLT) for spinopelvic fixation (SPF) uses a single U-shaped rod to restore the spinopelvic biomechanical integrity. The CLT method was designed to provide a non-rigid fixation, however this hypothesis has not been previously tested. Here, we establish a computational method to measure the deformation of the implant and characterize the bony fusion process based on the 6-year follow-up (FU) data.

**Materials and Methods:** Post-operative CT scans were collected of a male patient who underwent total sacrectomy at the age of 42 due to a chordoma. CLT was used to reconstruct the spinopelvic junction. We defined the 3D geometry of the implant construct. Using rigid registration algorithms, a common coordinate system was created for the CLT to measure and visualize the deformation of the construct during the FU. In order to demonstrate the cyclical loading of the construct, the patient underwent gait analysis at the 6th year FU. First, a region of interest (ROI) was selected at the proximal level of the construct, then the deformation was determined during the follow-up period. In order to investigate the fusion process, a single axial slice-based voxel finite element (FE) mesh was created. The Hounsfield values (HU) were determined, then using an empirical linear equation, bone mineral density (BMD) values were assigned for every mesh element, out of 10 color-coded categories (1st category = 0 g/cm^3^, 10th category 1.12 g/cm^3^).

**Results:** Significant correlation was found between the number of days postoperatively and deformation in the sagittal plane, resulting in a forward bending tendency of the construct. Volume distributions were determined and visualized over time for the different BMD categories and it was found that the total volume of the elements in the highest BMD category in the first postoperative CT was 0.04 cm^3^, at the 2nd year, FU was 0.98 cm^3^, and after 6 years, it was 2.30 cm^3^.

**Conclusion:** The CLT provides a non-rigid fixation. The quantification of implant deformation and bony fusion may help understate the complex lumbopelvic biomechanics after sacrectomy.

## Introduction

Sacral tumors are rare pathologies, and their management typically generates a complex medical problem (1). The most common primary sacral tumors are chordomas, representing 40% of all primary sacral neoplasms (2). Chordoma is a malignant mesenchymal tumor with notochordal origin (3) characterized by a high recurrence rate and a very limited response to non-surgical treatments. The surgical treatment is one of the most challenging fields in spine surgery because of the complicated anatomy of the sacral site. In most cases, only en-bloc surgical procedures, such as partial or total sacrectomy can guarantee optimal local control; but several problems such as bowel, bladder and sexual dysfunction; infection; massive blood loss; and spinopelvic instability can be associated with these surgeries (4, 5).

**Figure 1:**
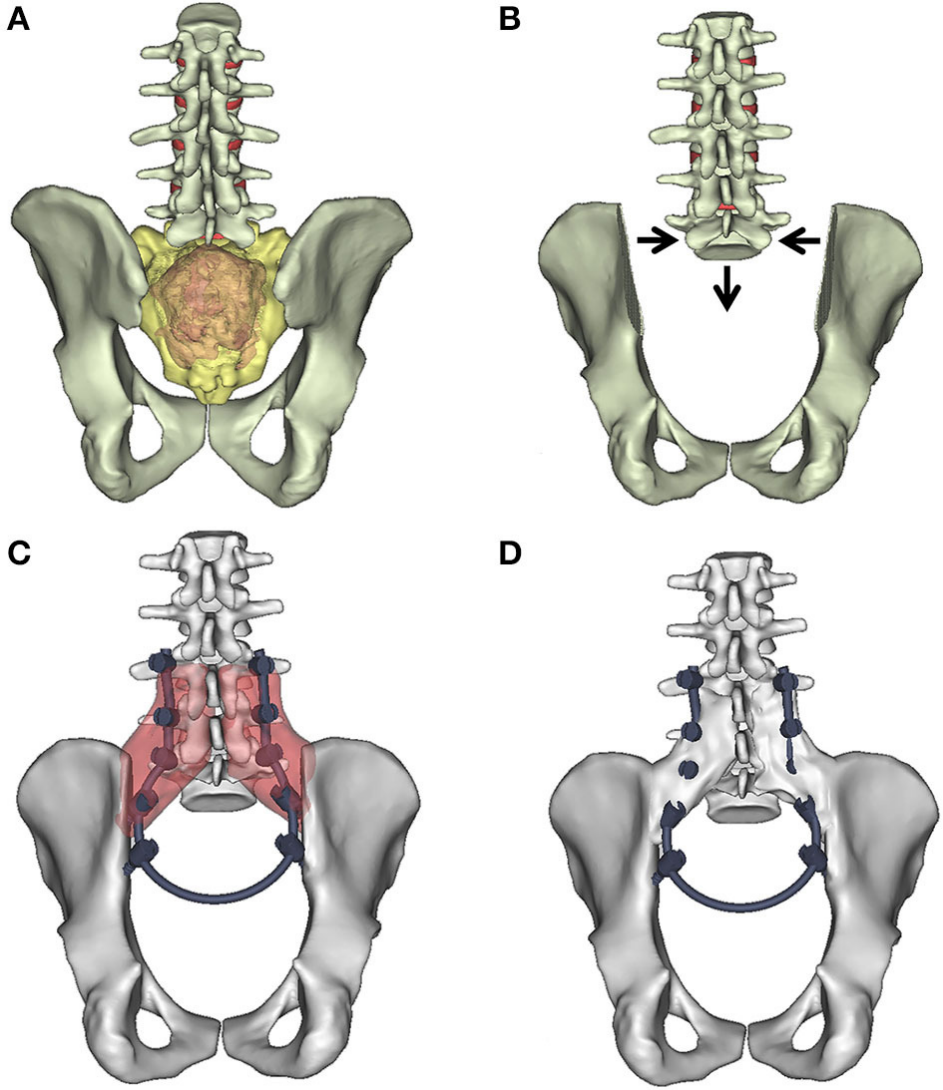
“Closed-Loop” lumbosacral reconstruction technique after total en-bloc sacrectomy. **(A)** Extended tumor mass affects the whole sacrum. (**B)** Geometrical change in the 3D geometry of the spino-pelvic junction after en-bloc total sacrectomy. The iliac bone is cut by an oscillating saw bilaterally; the medial cortical surface of the iliac bone is left on the specimen. The lumbosacral facet joints with the intervertebral discs are removed. The dural sac (together with the cauda equina) is cut through immediately below L5. The distance between the L5 vertebra and the iliac bone is reduced (direction of the arrows). **(C)** In the L3–5 vertebral body and bilaterally into the iliac bones, screws are inserted and connected with a single 5.5 mm diameter “U” shaped rod according to the patient's reduced **(C)** local dimensions and attached to the screws. The red areas mark the place for the artificial bone substitute, mixed with autologous bone graft. **(D)** The re-established connection between the lumbar spine and the pelvis. At the side of the graft (**D**) after 2 years bony fusion is expected.

Beyond the primary goal of the surgery (e.g., wide, en-bloc resection of the tumor mass), the optimal spinopelvic reconstruction, focusing on biomechanical stability and soft tissue restoration, is also indispensable (6). Several different techniques were developed for lumbopelvic stabilization after sacropelvic tumor resection (7). However, long-term follow-up data and comparative studies of the different techniques are rare or still missing. There is no gold standard, and relatively high complication rates (i.e., non-union, screw loosening, rod breakage) are reported with all reconstruction strategies (7, 8). The “en-bloc” resection of a sacral chordoma by performing a total sacrectomy with soft tissue and bony reconstruction, and lumbopelvic stabilization can be achieved with the closed-loop technique (1, 7). The technique uses a “U” shaped rod which is attached to the iliac and transpedicular screws to rebuild the spinopelvic connection (Figure 1). The closed-loop technique (CLT) was first introduced by Varga et al. (1), and it was adopted by others (9) and further developed (10).

The CLT method was designed to provide a non-rigid fixation, however this hypothesis has not been tested previously. Based on the hypothesis, implant deformation and continuous bone remodeling at the fusion site is expected. Here, we aimed to develop a generalizable method, based on patient-specific 3D geometries derived from a representative patient's computed tomography (CT) scans in order to investigate the permanent implant construct's contour deformation, and map the bony fusion process over a 6-year follow-up (FU). Long FU (more than 2 years) can provide evidence about the success of the reconstruction, if no implant-related failure occurred. In the case of deformity correction, the deformation of the implant construct (rod) has been investigated *via* contour deformation measurements (11–14). However, in the case of lumbopelvic reconstructions, the permanent contour deformation over the FU period has not been investigated so far.

## Methods

In this study, a clinical case is used to present a method for the evaluation of the implant construct's contour deformation in a lumbopelvic reconstruction, based on the postoperative (postop) CT scans *via* image processing methods (exp segmentation, rigid registration). The bony fusion was mapped in the CT scans by quantifying the Hounsfield unit (HU) values of the voxels in the region of interest and converted into bone mineral density (BMD) equivalent values.

### Clinical Case

The criteria for case selection were: the previous publication of the surgery, with video documentation and long (more than 2 years) FU, and the patient's ability to walk after surgery.

**Figure 2:**
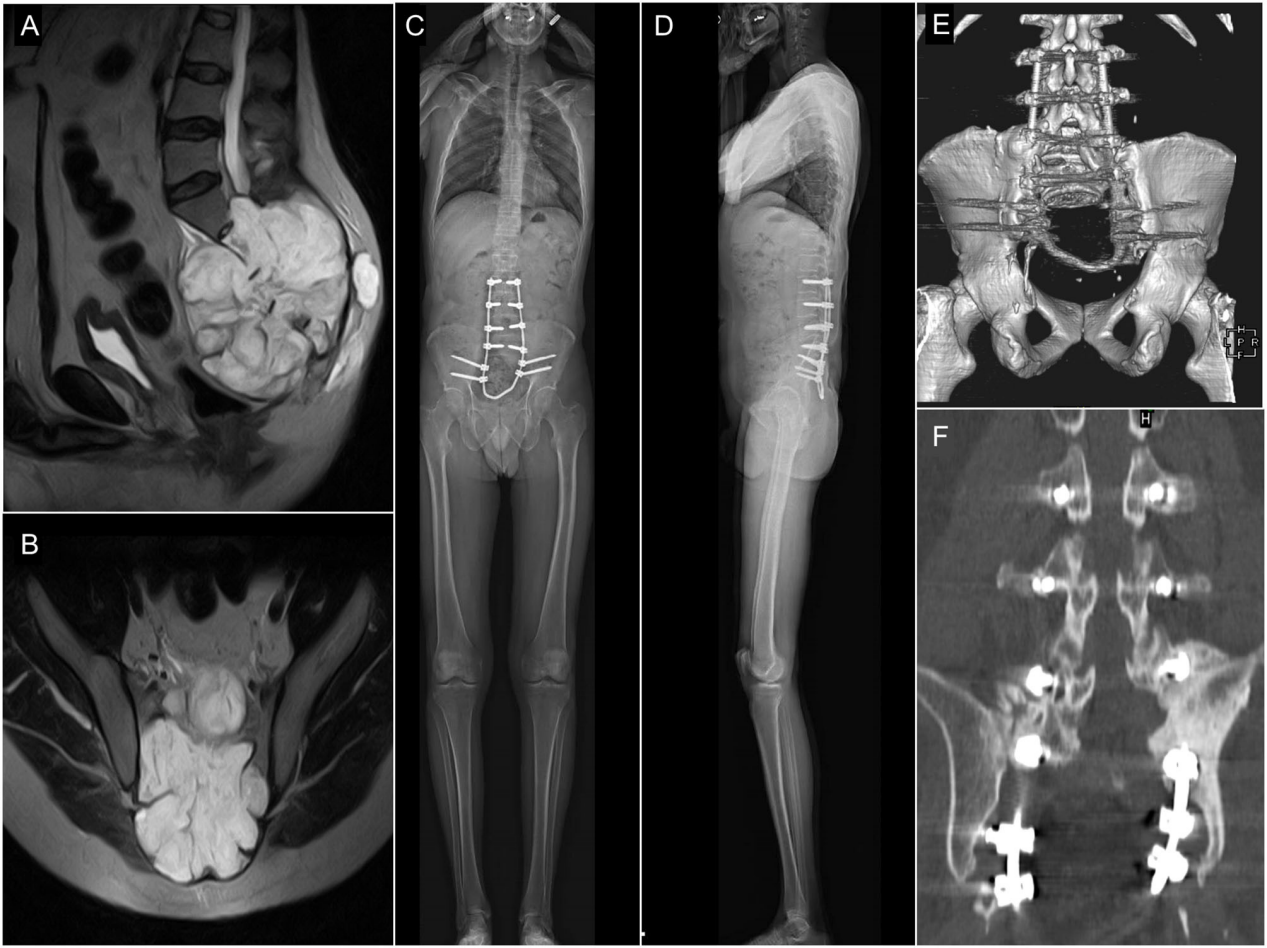
Pre- and postop imaging of a 42 years old male patient who underwent total en-block sacrectomy and received a Closed-Loop spino-pelvic reconstruction. **(A,B)** Preop T2, MRI images of a large sacral chordoma [**(A)** sagittal, **(B)** axial plane]. The extended tumor mass affected the whole sacrum with significant soft tissue extension to the retroperitoneum and cranially involving the paravertebral muscles. **(C,D)** Standing X-ray images of the patient at 6-month FU [**(C)** sagittal, **(D)** coronal plane]. **(E,F)** CT scan at 24-month FU period. Signs of bony fusion are visible between the L4 and L5 vertebrae and the iliac bone [**(E)** posterior view of the 3D rendered CT images; **(F)** coronal view at the fusion site].

The patient's (Figure 2) case and surgery were presented at the European Spine Journal, *Open Operating Theatre* (OOT) platform (15, 16). The 42-year-old male patient had mild and non-specific low back pain for 4–5 years. He had experienced minor problems with defecation and urination for 1 year, and a palpable lump had been observed for some months in the sacral region. Neurological examination showed normal motor and sphincter function but mild hypesthesia in the perianal region. Radiological examinations revealed an extended tumor mass affecting the whole sacrum with significant soft tissue extension to the retroperitoneum and cranial involvement of the paravertebral muscles as far as the L3 spinal level on the right side (Figure 2). Open biopsy-based histological examination confirmed the diagnosis of chordoma. Total “en-bloc” sacrectomy, combined with soft tissue and bony reconstruction, together with lumbopelvic stabilization (“closed-loop” technique, poly-axial titanium pedicle screw, with 5.5 mm diameter titanium rod, CD Horizon©LEGACYTM 5.5, Medtronic Sofamor Danek, Memphis, Tennessee, United States) was performed to remove the tumor *via* a posterior-only approach. Artificial bone substitute (ACTIFUSE^®^, Baxter International Inc., Deerfield, Illinois, United States) was placed between the L5 vertebral body and the iliac crest bilaterally, after refreshing and preparing well bleeding trabecular bony host surfaces (Figure 1C). The large defect of the body wall between the L5 vertebral body and the coccygeal ligamentous complex was covered by Dacron mesh (anchored to the bony landmarks: L5 vertebral body, tuber ossis ischii, and iliac bone). Finally, wound closure was performed by creating bilateral m. gluteus maximus rotational flaps. During surgery, the lumbosacral intervertebral disc was resected, and the dural sac (together with the cauda equina) was cut through immediately below the L5 origins. Cranial and ventral ligaments of the SI joints, and nerve roots (below the S1 segment) were both cut through bilaterally at the lateral aspect of the tumor (16). However, the patient was able to walk with crutches at the 3rd month FU, and without any assisting device at the 12th month FU. In order to quantify and evaluate the gait of the patient at the 6th year FU, gait analysis was performed, see Supplementary Material (Supplementary Study I).

### Postoperative Computed Tomography Scan Acquisition

Analysis of retrospectively collected postop CT data was performed. The study was approved by the National Ethics Committee of Hungary, the National Institute of Pharmacy and Nutrition (reference number: OGYÉI/163-4/2019). Informed consent was obtained from the participant. The data set consisted of 12 CT scans, covering a 6-year FU period (Table 1). The CT scans were performed with the same CT machine (Hitachi Presto, Hitachi Medical Corporation, Tokyo, Japan) with an intensity of 225 mA and a voltage of 120 kV. CT slice distance and resolution were 3.75 mm and 512 by 512 pixels, respectively. The data were exported from the hospital PACS in DICOM file format. To comply with the ethical approval for patient data protection, deidentification of the DICOM data was performed using the freely available Clinical Trial Processor software (Radiological Society of North America, https://www.rsna.org/ctp.aspx) (17).

**Table 1:** Retrospectively collected CT scans.

Postop years	1					2		3	4	5	6	
Number of CT scan	1	2	3	4	5	6	7	8	9	10	11	12
Days after surgery	7	34	83	138	250	446	656	1027	1384	1734	1937	2112

*CT, computed tomography*.

### Image Processing, 3D Geometry Definition

**Figure 3:**
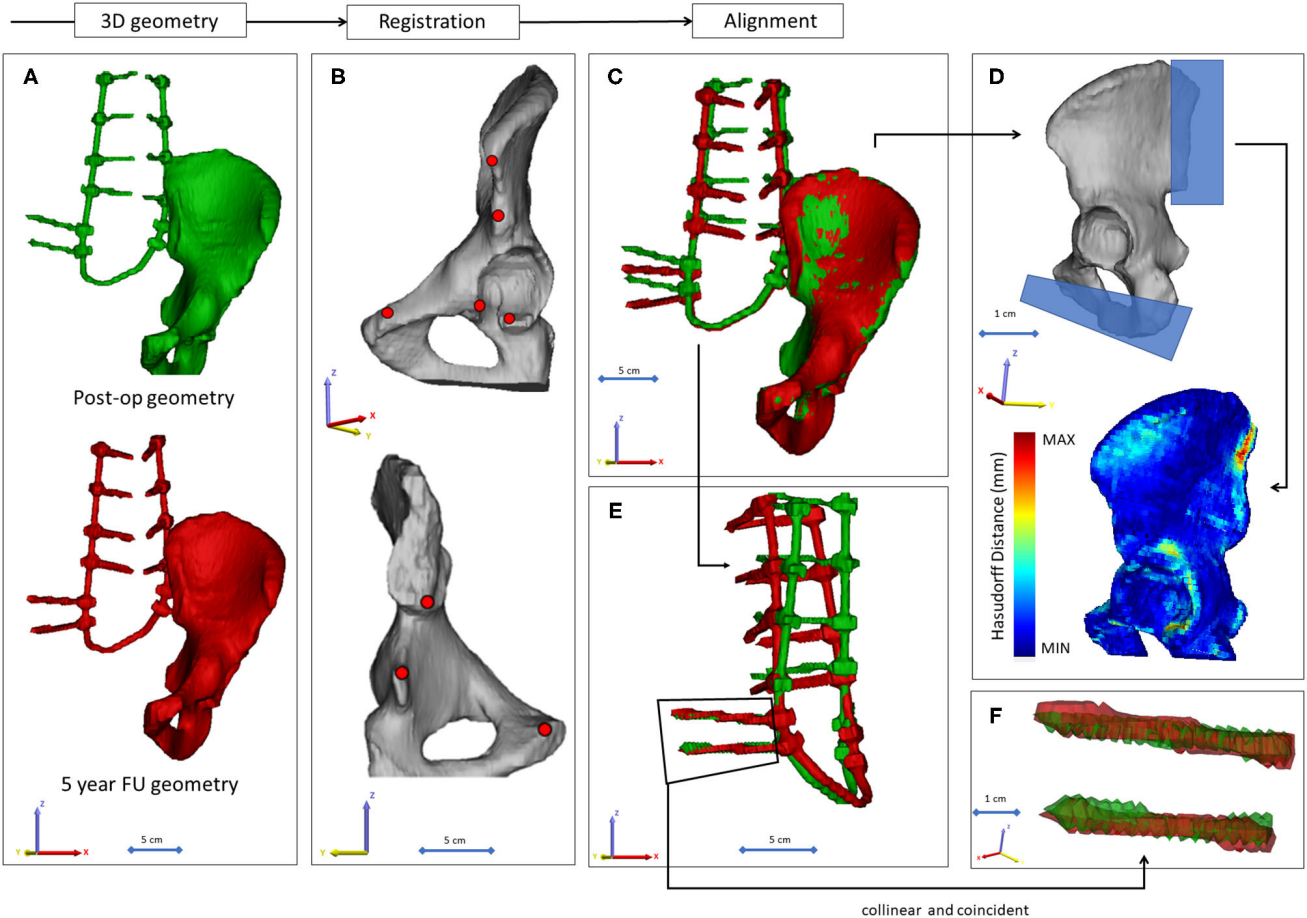
Post-op CT scan-based geometry definition and alignment. **(A)** Thresholding based segmentation was performed on the postop CT scans in order to define the left pelvic bone and the implant construct. **(B)** Eight points corresponding to anatomical landmarks were used for the simultaneous registration of the pelvic bone and implant construct geometry. **(C)** Every postop pelvic bone + implant construct geometry was registered to the first postop geometry. **(D)** The Hausdorff Distance was used as a metric for the alignment accuracy evaluation. Geometrical reduction of the caudal and posterior parts of the registered pelvic bones was performed. **(E)** The trans-iliac screw bodies geometry overlapped after the pelvic bone registration. **(F)** The axes of the iliac screws were considered collinear and coincident.

The 3D geometry of the closed-loop implant and the left pelvic bone were defined in every CT data set. No radiological sign of implant failure (screw loosening, screw or rod breakage, screw/rod disconnection) was registered in the 12 CT scans. The segmentation process was performed on the CT images (18) in the Mimics^®^ image analysis software (Mimics Research, Mimics Innovation Suite v21.0, Materialise, Leuven, Belgium) (Figure 3A). The left pelvic bone was isolated, and then the implant geometry was separated. The resulting masks (groups of voxels) were homogeneously filled by preserving the outer contour of the geometrical border in 2D. From the masks, a triangulated surface mesh was automatically generated for the pelvic bone and the implant construct (Figure 3A). To evaluate the accuracy of the segmentation process, Dice Similarity Index (DSI) (19, 20) was calculated in each case. DSI values range between 0 and 1, with 1 denoting a perfect match. The implant geometry and the pelvic bone geometry were segmented 12 times by two investigators: I_1_ and I_2._ The DSI was calculated by comparing the segmentation of I_2_ to I_1_ (21).

### Alignment of the Implant Construct Geometries

To determine the implant construct's contour deformation, the 12 segmented (I_1_) implant geometries were aligned with the pelvic bone in the same coordinate system. The left pelvic bone in the first postop CT scan was used as a reference geometry. A control points-based rigid registration algorithm was used in Mimics^®^. The 8 control points corresponded to easily identifiable anatomical landmarks on the left pelvic bone (Figure 3B). During registration, the implant construct moved together with it's corresponding pelvic bone (Figure 3C). At the fusion site, after the alignment, a symmetrical geometry reduction was performed using cuboid subtraction, in order to exclude the geometrical difference (Supplementary Figure 2). A second geometry subtraction was performed for all pelvic bone geometries at the ischial ramus, where the last axial CT slice ended in the first postop scan (Figure 3D; Supplementary Figure 2). The uniformly reduced pelvic bone surface geometry was considered constant. To evaluate the accuracy of the registration and alignment procedure, and to demonstrate that the reduced pelvic bone surface geometry can be considered constant, Hausdorff Distance (HD) was measured with the MeshLab 1.3.2 software's (22) (http://www.meshlab.net) Metro tool (23) (Figure 3D) at the level of the aligned pelvic bones. The alignment of the 12 geometries was performed by the first investigator (I_1_), and the HD measurements were performed on each postop CT scan, where the geometries derived from the 2nd−12th CT scans were compared to the first postop scan-based geometry. After registration of the pelvic bones, the trans-iliac screw bodies' geometries overlapped. The axes of the iliac screws were collinear and coincident (Figures 3E,F). To test this hypothesis, HD values were calculated for the screw bodies by comparing the geometries to the first postop CT scan geometry after the alignment.

### Implant Deformation Measurements

**Figure 4:**
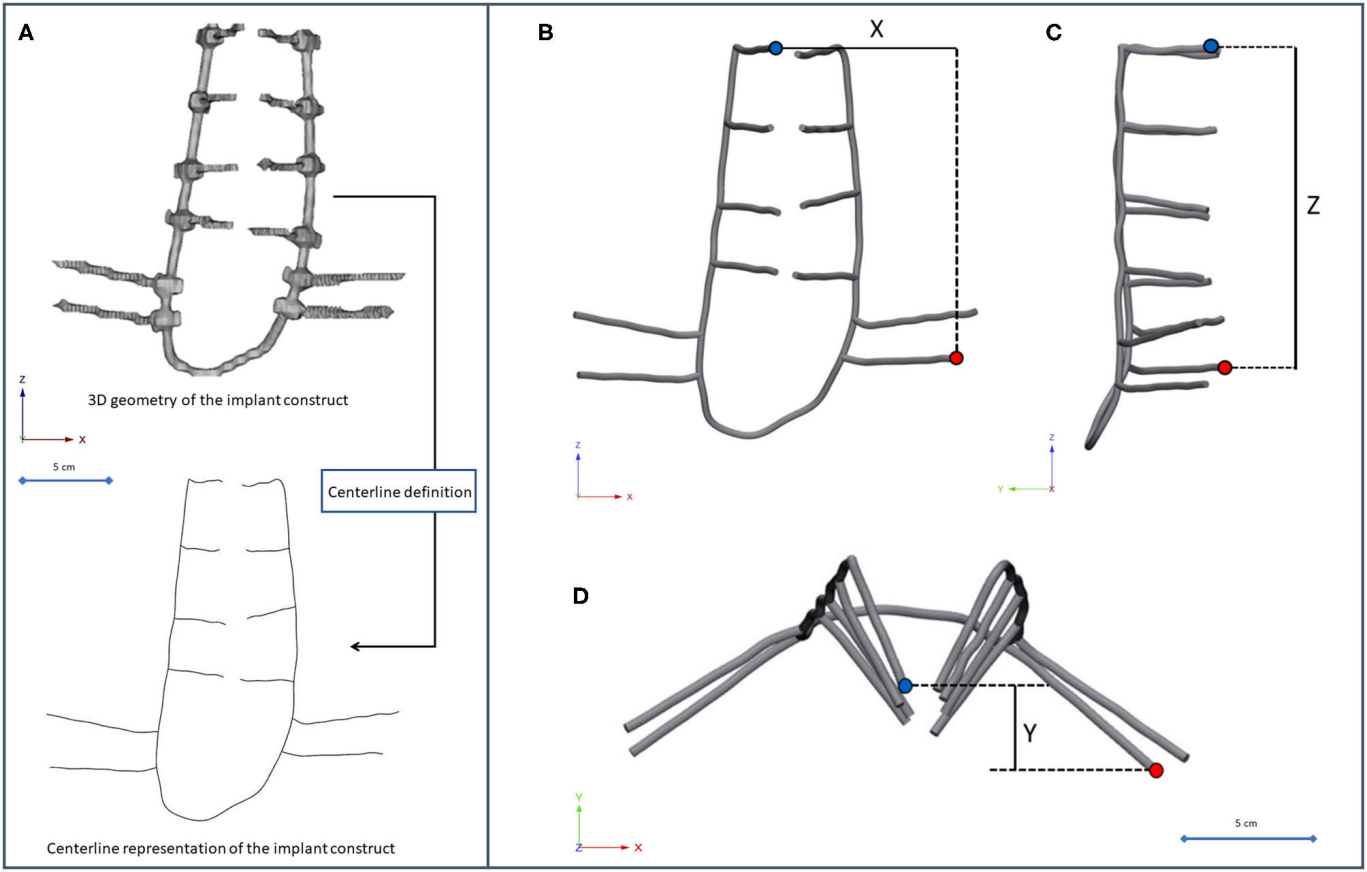
Implant construct geometry simplification and deformation measurement. **(A)** The segmented geometry of the implant construct was considered a tubular structure, the centreline of the geometry was defined. **(B–D)** A fixed point (red dot) was selected in the centreline corresponding to the tip of the caudal trans iliac screw, and a mobile point (blue dot) corresponding to the L2 right pedicle screw tip. The distance between the points was determined **(B)** in the coronal plane (X_d_), **(C)** sagittal plane (Z_d_), **(D)** axial plane (Y_d_).

The implant construct geometry was considered a tubular structure, and the centreline of the geometry was defined with Mimics^®^ (Figure 4A). A mobile point, corresponding to the L2 right pedicle screw tip, and a fixed point, corresponding to the tip of the caudal iliac screw, were selected in the centreline. The distances between the points were measured in three anatomical planes (Figures 4B–D) using 3-matic^®^ (Mimics Innovation Suite v21.0, Materialise, Leuven, Belgium). The segmentation of the implant construct, the centreline definition, and the distance measurement in the three planes were performed by three investigators (I_1_, I_2_, I_3_) at two different time points (T_1_, T_2_). In order to test the repeatability and reliability of the measurements, three-dimensional distance (3D_d_) was calculated based on the X_d_ (coronal plane), Y_d_ (axial plane), and Z_d_ (sagittal plane), using the formula ${\text{3D}}_{\text{d}}=\sqrt{X_{d}^{2}+Y_{d}^{2}+Z_{d}^{2}}$.

### Mapping of the Bony Fusion

**Figure 5:**
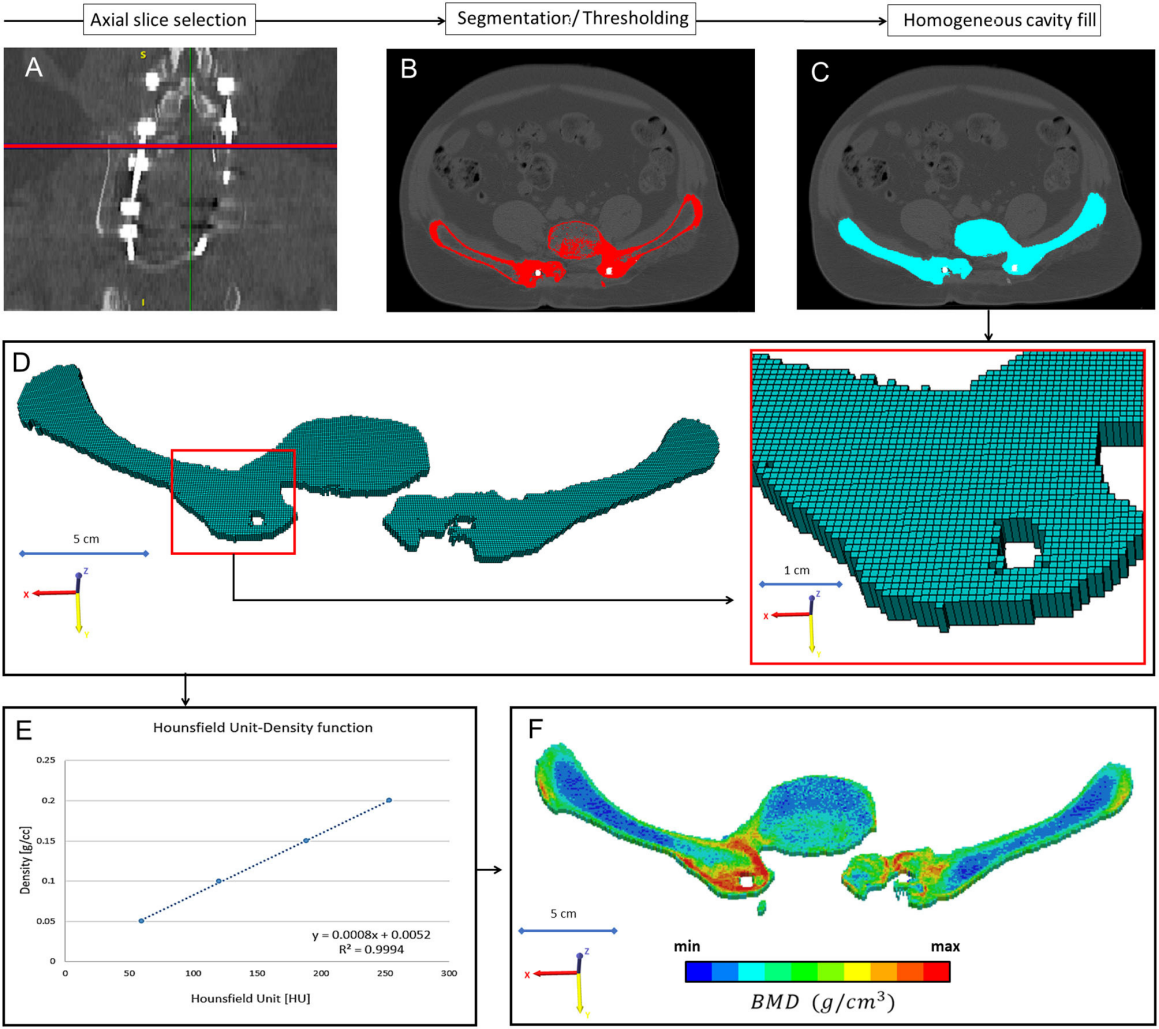
Evaluation of the bony fusion process between the L5 vertebra and the two iliac bones. **(A)** From all the 12 CT scans the same region of interest (midplane between the right L4 and L5 pedicle screw) an axial slice was selected. **(B)** The bony elements were segmented in the selected slice. **(C)** A homogeneous mask was created corresponding to the segmented bony elements. **(D)** A voxel-based FE mesh was created based on the segmented mask. **(E)** A linear relationship was used to assign the bone mineral density values for the corresponding Hounsfield values. **(F)** In the voxel-based FE mesh, every voxel was coded with a color code corresponding to the BMD.

In every CT scan, from the same anatomical region (midplane between the right LIV and LV pedicle screw), a single axial slice was selected (Figure 5A) as a region of interest (ROI). The bone tissue was segmented based on a thresholding algorithm (left and right iliac bone, and LIV vertebra) (Figure 5B). The masks' internal part contained voxels representing adipose tissue (BMD close to 0). From this mask, a voxel-based finite element (FE) mesh was created with Mimics^®^ (Figures 5C,D). CT scans from the FU were acquired without a densitometric calibration phantom. From the institutional PACS database, QCT scans were selected with the same acquisition protocol and machine (Table 1). The dates of the scans were selected to be in the same months as the postop CT scans. The male subjects also had similar body mass indices (BMI = 28 ± 2) as the presented patient (BMI = 28). The Hounsfield unit (HU) values of the QCT images were converted into BMD equivalent values by using a densitometric calibration obtained with an inline phantom (Hitachi Presto, Hitachi Medical Corporation, Tokyo, Japan) consisting of five cylindrical insertions with known mean equivalent BMD values (0, 0.5, 0.1, 0.15, and 0.2 g/cm^3^). Based on the 12 QCTs, a mean conversion curve was defined and assumed to be linear [BMD = ρQCT = a + b ^*^ HU, where ρQCT (g/cm^3^) is bone density] according to prior studies (24, 25) (Figure 5E). In the voxel-based FE mesh, every element was color-coded, using 10 colors, corresponding to BMD values as shown in Figure 5F. The changes in the distribution of the sets of FE mesh voxel elements over the FU were analyzed. The volumes of the voxels in every BMD category were calculated (voxel dimensions ^*^ number of elements) and visualized using a 3D surface plot (**Figure 9**) created with SigmaPlot 12 (SSI, San Jose, California, United States).

## Statistical Analysis

The reliability and repeatability of our method was tested in order to demonstrate the reproducibility of the study. Due to the small sample size, we used non-parametric tests. Inter-rater (I_1_ vs. I_2_ vs. I_3_) reliability was determined by intraclass correlation coefficient (ICC) estimates, and their 95% confidence intervals (CI) were calculated based on a mean-rating (*k* = 3), absolute-agreement, 2-way mixed-effects model. Intra-rater (I_1_T_1_ vs. I_1_T_2_, I_2_T_1_ vs. I_2_T_2_, I_3_T_1_ vs. I_3_T_2_) reliability was determined by ICC estimates, and their 95% confidence intervals were calculated based on a single measurement, absolute-agreement, 2-way mixed-effects model. The relationships between the implant deformation in the anatomical planes and the number of postop days, and between the volume change in the different BMD categories were analyzed using the Spearman's rank correlation (**Figures 8**, **10**). The interpretation of the correlation was based on the work of Evans et al. (26). All statistical tests were performed with SPSS^®^ Statistics 23 software (SPSS Inc, Chicago, IL).

## Results

### Evaluation of the Segmentation Procedure

The obtained DSI values (Supplementary Table 1) for the implant construct geometries were very high 0.97 ± 0.02 (*n* = 12) as well as for the pelvic bone 0.96 ± 0.05 (*n* = 12) and showed negligible variance, indicating high accuracy of the segmentation method for all segmented geometries (27).

### Alignment Evaluation

**Figure 6:**
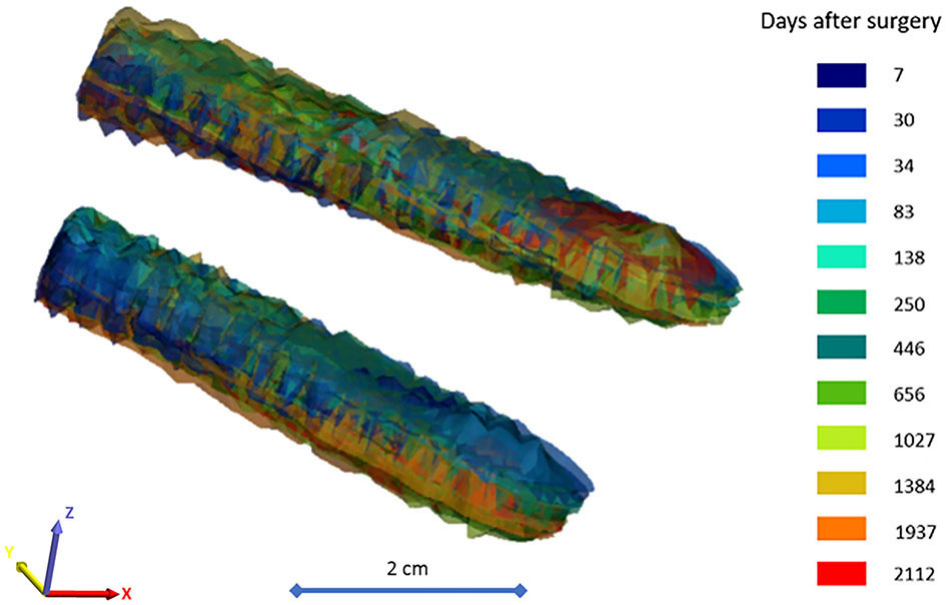
Geometric overlap of the body of the iliac screws after the alignment process. The 12 postop CT scan-based surface mesh representing the iliac screw bodies are color-coded corresponding to the scale bar (color = CT scan session + number of days after surgery). The surfaces mesh is visualized with 75% transparency.

A mean HD of 0.63 ± 0.14 mm was obtained (Supplementary Table 2) from the reduced pelvic bone geometries. The HD determined for the iliac screw bodies had a mean value of 0.95 ± 0.10 mm (Supplementary Table 3). These values are considered by the field to be indicative of adequate fitting (28). The colinear and coincident position of the iliac screw axes, and the geometric overlap of the bodies were visualized in Figure 6. The figure and the HD values (Supplementary Table 3) demonstrate that the screw body does not deform or change its position in the common coordinate system. Theoretically, any point in these two screw body geometries can be used as a reference point in a measurement process. The right pelvic bone could be selected for the analysis as well, with the right iliac screw bodies, as demonstrated in Supplementary Study II.

### Implant Deformation

**Figure 7:**
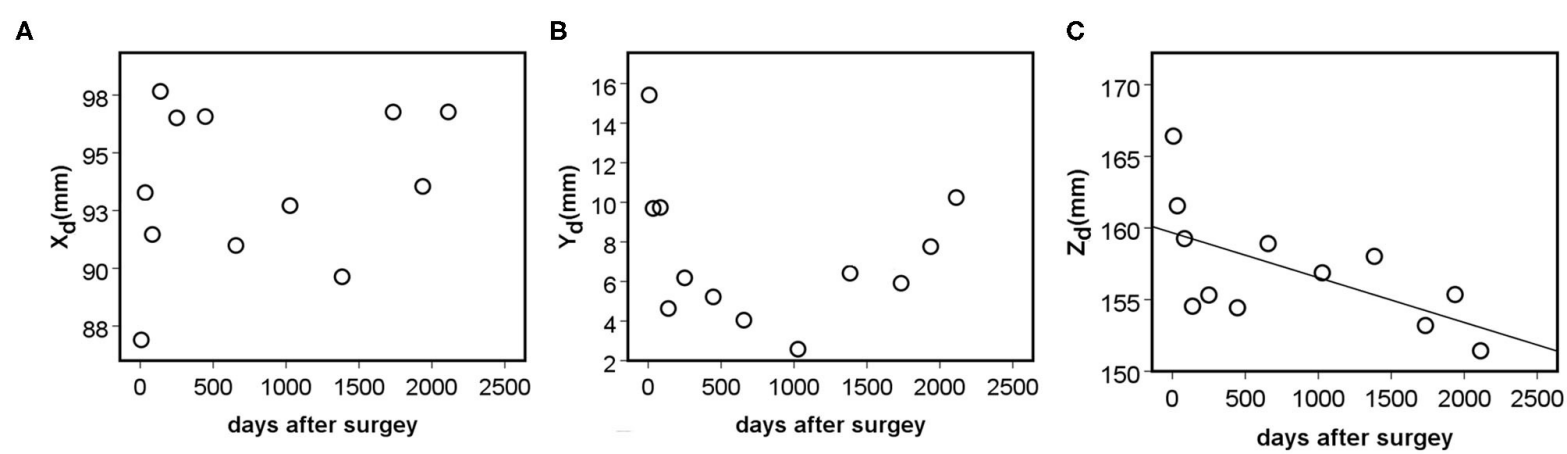
Association between the distance of the mobile (L2 right pedicle screw tip) point from the fixed point (left, caudal trans iliac screw tip) in the anatomical planes, and the number of days after surgery (DAS). **(A)** Non-significant positive, moderate correlation was found between the X_d_ (frontal plane) and DAS (ρ = 0.336, *p* = 0.286). **(B)** Non-significant, negative, weak correlation was found between the Y_d_ (axial plane) and DAS (ρ = −0.182, *p* = 0.572). **(C)** Significant, negative, and strong correlation was found between the Z_d_ (sagittal plane) and the number of days after surgery (ρ = −0.664, *p* = 0.018).

The implant deformation measurements are summarized in Supplementary Tables 4, 5, and 6. The mean displacement relative to the first postop CT scan was ΔX_d_ = 7.27 ± 2.80 mm in the frontal plane, ΔY_d_ = 8.24 ± 2.51 mm in the coronal plane, and ΔZ_d_ = 10.15 ± 2.97 mm in the sagittal plane. To test the accuracy and reproducibility of these measurements, inter- and intra-rater reliability tests were performed by calculating the intraclass correlation coefficient (ICC) based on the 3D_d_ values, presented in Table 2. The ICC values show excellent reliability, with the exception of the I_1_T_1_ vs. I_1_T_2_ intra-rater reliability, where the ICC was 0.768, which corresponds to good reliability (29). The association between the average X_d_/Y_d_/Z_d_ measurement and the number of days after surgery is shown in Figure 7. The implant construct's deformation can be registered in the anatomical planes over the postop FU period. However, the deformation was only significant in the sagittal plane, showing a strong negative correlation between the Z_d_ and the number of days after surgery (ρ = −0.664, *p* = 0.018). This result demonstrates the forward bending tendency of the construct.

**Table 2:** Results of the two-way mixed absolute agreement calculation for ICC.

	**ICC**	**95% confidence interval**	
		**Lower bound**	**Upper bound**
**Intra-rater reliability**			
I_1_T_1_ vs. I_1_T_2_	0.768	0.362	0.928
I_2_T_1_ vs. I_2_T_2_	0.980	0.934	0.994
I_3_T_1_ vs. I_3_T_2_	0.949	0.838	0.985
**Inter-rater reliability**			
I_1_ vs. I_2_ vs. I_3_	0.980	0.948	0.994

*ICC, intraclass correlation; I, investigator; T, time point*.

### BMD Mapping at the Fusion Site

**Figure 8:**
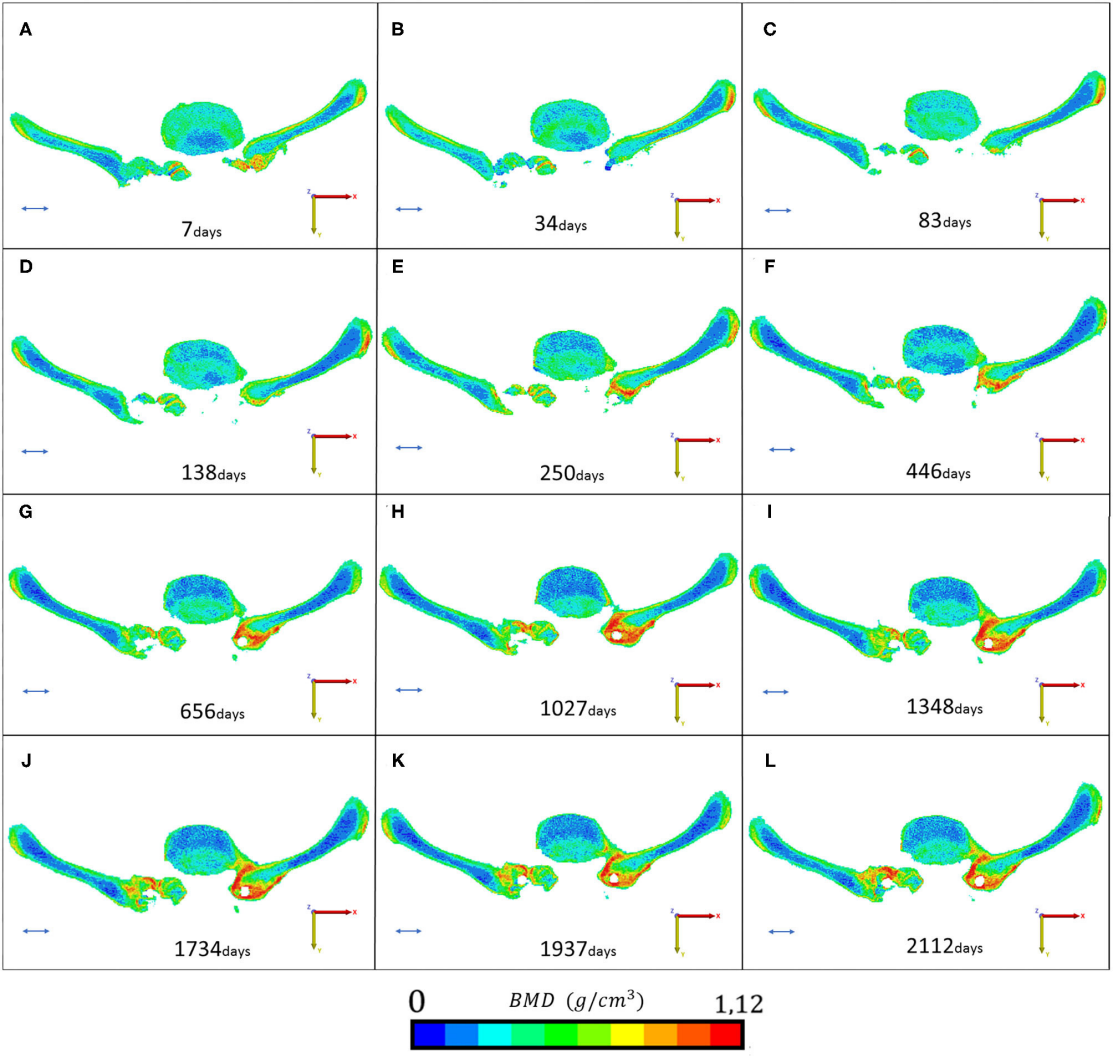
Mapping of the fusion and remodeling process. (**A**–**L)** Represents the region of interest for the 12 postop CT scans (from 7 to 2,112 days). The BMD values are represented in 10 color codes from 0 to 1.12 g/cm^3^ on an RGB scale. Red color represents the strongest bone tissue. The provided scale bar's length is 2 cm.

**Figure 9:**
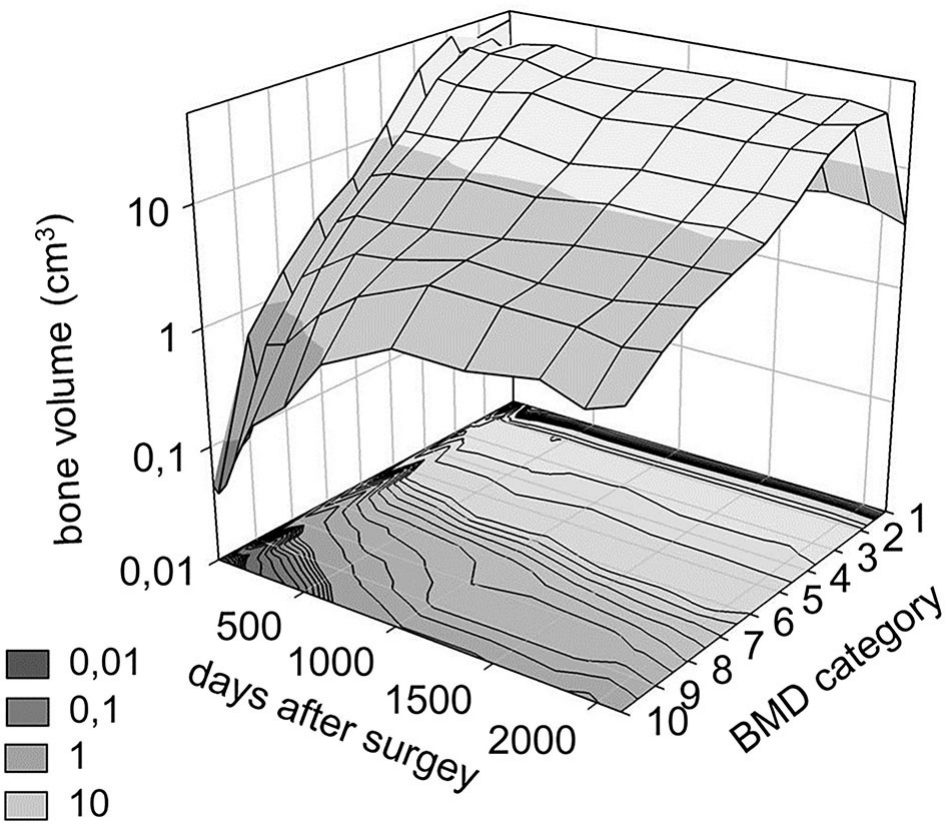
Distribution of bone volume in the 10 BMD category over the follow up period. The bone volume is defined using the FE mesh voxel dimensions. The BMD categories from 1 to 10 correspond to the color code from (8) (1st category = 0 g/cm^3^, 10th category 1.12 g/cm^3^).

**Figure 10:**
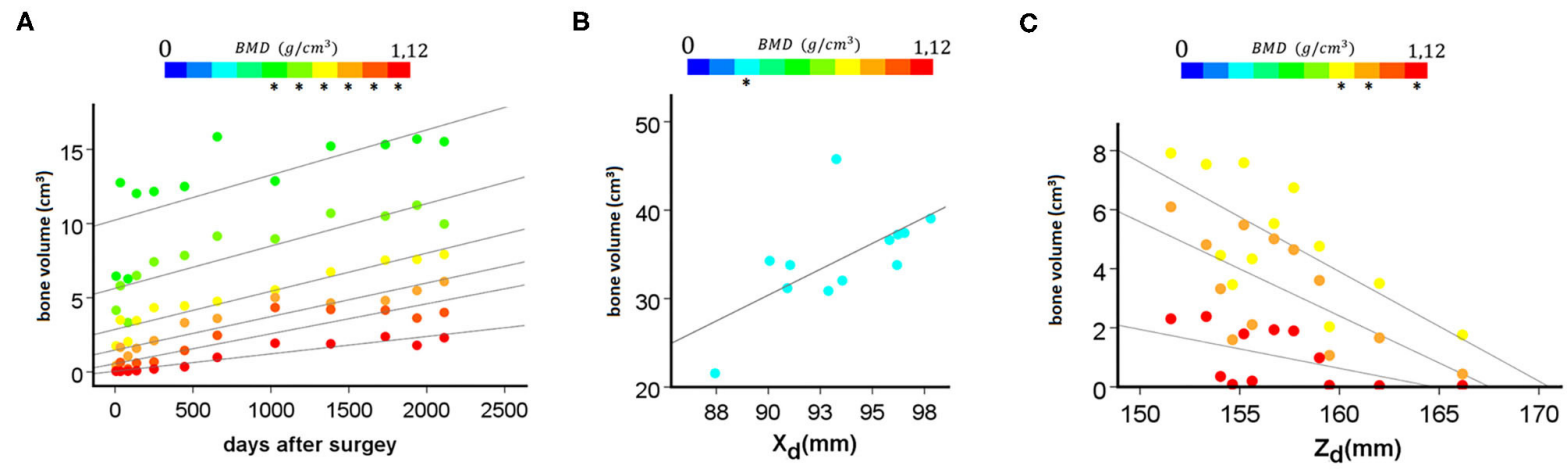
Association between BMD categories' volumetric change and the days after surgery and the distance of the mobile (L2 right pedicle screw tip) point from the fixed point (left, caudal trans iliac screw tip) in the anatomical planes. **(A)** Significant positive, very strong correlation (ρ > 0.800, *p* < 0.050) was found between the highest 6 BMD categories (marked with “*”) and the days after surgery. **(B)** Significant positive, strong correlation was found between the X_d_ (frontal plane) and the third BMD category (ρ = 0.678, *p* = 0.015). **(C)** Significant negative, strong correlation (ρ > 0.600, *p* < 0.050) was found between 3 high value BMD categories (marked with “*”) and the Z_d_ (sagittal plane) measurements.

The bone material density distribution in the region of interest for the fusion process was measured over the FU period (Figure 8, Table 3). The color map captures the bone remodeling process in the ROI. After the second year FU, a solid fusion was detected between the lumbar spine, the L4 vertebra and the two iliac bones. However, due to the cyclical loading, the bone remodeling represented by the change in the element distribution in the color-coded BMD categories continued. The change in the volume of the BMD categories over the days after surgery is presented in Figure 9. The obtained 3D contour plot demonstrates an increase in the high BMD category volume after the second year FU. The association between the 10 BMD categories' volumetric change and the days after surgery was significantly positive, with very strong associations (ρ > 0.800, *p* < 0.050) in the highest 5 BMD categories (Figure 10A). From the 10 BMD categories, no significant correlation was found between volumetric change and Y_d_ (axial plane). In the frontal plane (X_d_), only for the third BMD category was found to have significant, strong correlation (ρ = 0.678, *p* = 0.015) (Figure 10B). Significant negative correlation (ρ > −0.600, p < 0.050) was found only between 3 high-value BMD categories and the Z_d_ (sagittal plane) measurements (Figure 10C).

**Table 3:** Volumetric change of the voxel groups corresponding to the BMD categories.

**No**.	**Days after surgery**	**Volume of the different BMD category's during the FU (cm**3**)**									
											
1	7	41.70	36.36	21.55	9.59	6.46	4.16	1.76	0.43	0.09	0.05
2	34	0.41	17.96	45.77	29.79	12.76	5.81	3.50	1.66	0.64	0.04
3	83	17.96	36.52	33.79	13.93	6.27	3.33	2.04	1.07	0.21	0.05
4	138	1.10	31.22	39.06	26.28	12.03	6.50	3.46	1.60	0.60	0.08
5	250	2.50	36.33	37.25	25.14	12.16	7.42	4.34	2.11	0.68	0.20
6	446	3.43	40.58	37.42	25.22	12.50	7.85	4.45	3.32	1.45	0.35
7	656	3.04	36.20	31.18	28.78	15.85	9.15	4.76	3.61	2.47	0.98
8	1,027	3.22	38.04	30.87	23.40	12.87	8.97	5.53	5.01	4.34	1.94
9	1,384	3.89	39.48	34.27	25.99	15.22	10.69	6.74	4.65	4.21	1.90
10	1,734	4.73	38.00	33.79	25.83	15.32	10.51	7.54	4.81	4.17	2.38
11	1,937	4.38	39.47	32.04	26.55	15.70	11.24	7.59	5.49	3.63	1.79
12	2,112	3.45	35.72	36.63	27.69	15.52	9.97	7.92	6.10	4.01	2.31
**BMD category's (g/cm**3**)**											
		0.00	0.12	0.25	0.37	0.50	0.62	0.75	0.87	1.00	1.12
											

*BMD, bone mineral density; FU, follow up; No, Number of CT scans*.

## Discussion

In the presented study, we investigated the hypothesis that the CLT method provides a non-rigid fixation of the lumbopelvic junction after sacrectomy. We aimed to develop a method for the investigation of implant deformation and bony fusion using the postop CT scans collected over the 6-year FU period of a patient who underwent sacrectomy and closed-loop reconstruction. We were able not only to demonstrate the non-rigidity of the construct by measuring the geometrical deformation over the FU period, but to map the bony remodeling at the fusion site (lumbar spine and two iliac bones) as well. Significant correlation was found between deformation in the sagittal plane and postop days, denoting the forward bending tendency of the construct. The closed-loop reconstruction technique can provide excellent locomotor outcomes after total sacrectomy; a similar result was demonstrated by Smith et al. (30). The fact that the presented patient was able to walk resulted in a periodic cyclical loading of the construct. Clark et al. (31) compared three spinopelvic reconstruction techniques under gait-simulating fatigue loading and sagittal alignment failure on cadaveric specimens. Despite the complex gait-like loading experiment, due to its limitations (cadaveric specimen), it does not take into consideration the bony fusion process, only focusing on the primary stability of the construct. We introduced the centrelines to avoid artifacts in the CT scans, even so, the resolution of the clinical CT affects the straightness of the centreline. In the future, after applying the method to a larger patient cohort, we plan to perform a curvature analysis methodologically similar to Hay et al.'s work (32), in which the centreline would be a technical advantage.

According to Frost's *mechanostat* theory (33, 34), bone growth and bone loss are stimulated by the local mechanical elastic deformation of bone. Effects of the implant construct's stiffness on the healing of fractures in the case of long bones stabilized by internal fixation has been widely investigated (35, 36), and it is known that constructs that are too stiff lead to non-union (37–40). In the case of posterior spinal fixation, the stiffness of implant rods (titanium alloys, stainless steel, cobalt-chromium–based alloys) can differ (13). Load sharing that occurs with the use of spinal implants results in a decreased load and thus reduced strain throughout the stabilized vertebral body, which leads to bone mineral density loss (13, 41).

Based on our results, the correlation between the high-value BMD categories and the forward bending tendency of the construct highlights the importance of rod stiffness (exp. diameter, material: cobalt-chrome or titanium) in the fusion process. Implant deformation *in vivo* due to upper body weight and muscle forces is rather well-known in cases of deformity correction. Several biomechanical studies have already investigated implant failure, mainly due to upper body flexion, especially in adult spine deformities and unbalanced patients (42–44). However, it has not been investigated in lumbopelvic reconstructions. The developed method showed high accuracy and repeatability.

The quantification of bone formation uses the voxel dimensions and the Hounsfield values of the voxel-based FE element mesh in the regions of interest. The application of the mapping method on a large patient group would be desirable for other reconstruction techniques as well. The optimal, expert opinion-based, consensual reconstruction technique is not currently defined. The retrospective investigation of different methods based on CT scans over long FU periods (≥2 years) would be important for a better understanding of these complex surgical problems and the development of new solutions. The data obtained *via* this method could be used for the validation of FE models by identifying the regions of the construct where the highest deformation occurred during the FU, and by comparing the effect of the construct's stiffness/rigidity on the fusion process (45). Nevertheless, the data collected with the presented method could be used in implant design, especially for 3D printed patient-specific solutions (46).

We would like to highlight the limitations of our study. Three months were dedicated to the project. The study was designed as a “method” article, however, implementation on a larger patient cohort would be desirable. The study only reported on the distance between a specific point of the implant construct and the fixed iliac screw bodies. Based on this result, it is not possible to distinguish the mechanical factors involved (axial/shear load, sagittal/lateral bending, or axial torsion) in the permanent deformation. Due to the retrospective nature of the study, it was not possible to perform a repeatability test at the same follow-up time after repositioning of the patient to assess the accuracy in the BMD assessment or the construct contour deformation measurement.

## Conclusion

The presented clinical image analysis-based computational method (segmentation, rigid registration, BMD assessment at the voxel level based on HU values) can provide accurate information about the implant construct's deformation after sacrectomy, following reconstruction with the closed-loop technique. We recommend the application of our measurement method for the scientific and clinical analysis of other surgical procedures as well, and other clinical scenarios where large constructs are needed, such as idiopathic or degenerative deformity corrections, growing rods systems, etc.

The BMD mapping at the fusion site may help in the future to evaluate the effect of the implant's (rod) diameter, or material, (titanium vs cobalt-chromium) on the fusion process.

The identification of regions where the constructs undergo the highest deformation may be useful in the surgical planning and in implant-related failure prevention.

## Data Availability Statement

The original contributions presented in the study are included in the article/Supplementary Material, further inquiries can be directed to the corresponding author.

## Ethics Statement

The study was approved by the National Ethics Committee of Hungary, the National Institute of Pharmacy and Nutrition (reference number: OGYÉI/163-4/2019). The patients/participants provided their written informed consent to participate in this study. Written informed consent was obtained from the individual(s) for the publication of any potentially identifiable images or data included in this article.

## Author Contributions

PE, DL, PV, and AL contributed to the conception and design of the study. PE, MT, FB, and JF did the CT based image analyses and the visualizations. The gait assessment was performed by JF, GS, and TT. PV and AL performed the surgery described in this case report. PE wrote the first draft of the manuscript and prepared the pictures together with MT. All authors contributed to manuscript revision, read and approved the submitted version.

## Funding

The project leading to the scientific results was supported by the Hungarian Scientific Research Fund Grant, Budapest, Hungary (Award Number: OTKA FK123884) and by the European Commission (766012-SPINNER–H2020-MSCA-ITN-2017), and by the Doctoral Student Scholarship Program of the Co-operative Doctoral Program of the Ministry of Innovation and Technology, Hungary, financed from the National Research, Development and Innovation Fund (C1014064). The financial support from these funding bodies are gratefully acknowledged.

## Conflict of Interest

The authors declare that the research was conducted in the absence of any commercial or financial relationships that could be construed as a potential conflict of interest.

## Publisher's Note

All claims expressed in this article are solely those of the authors and do not necessarily represent those of their affiliated organizations, or those of the publisher, the editors and the reviewers. Any product that may be evaluated in this article, or claim that may be made by its manufacturer, is not guaranteed or endorsed by the publisher.
