# Supplementary material for: A Novel Three-Dimensional Computational Method to Assess Rod Contour Deformation and to Map Bony Fusion in a Lumbopelvic Reconstruction After En-Bloc Sacrectomy
Source: Front Surg. 2022 Jan 5;8:698179. doi: 10.3389/fsurg.2021.698179 (PMC8766313; doi:10.3389/fsurg.2021.698179)
Supplement: Supplementary file 1 [file Data_Sheet_1.pdf]

A novel three-dimensional computational method to assess rod contour deformation and to map bony fusion in a lumbopelvic reconstruction after en-bloc sacrectomy

Supplementary Materials

Peter Endre Eltes[1,2*], Mate Turbucz[1,3*], Jennifer Fayad[1,4], Ferenc Bereczki[1,3], György Szőke[5], Tamás Terebessy[5], Damien Lacroix[6], Peter Pal Varga[7], Aron Lazary[2,7]

1. In Silico Biomechanics Laboratory, National Center for Spinal Disorders, Buda Health Center, Budapest, Hungary
2. Department of Spine Surgery, Semmelweis University, Budapest, Hungary
3. School of PhD Studies, Semmelweis University, Budapest
4. Department of Industrial Engineering, Alma Mater Studiorum, Universita di Bologna, Bologna, Italy
5. Department of Orthopaedics, Semmelweis University, Budapest, Hungary
6. INSIGNEO Institute for in Silico Medicine, Department of Mechanical Engineering The University of Sheffield, UK
7. National Center for Spinal Disorders, Buda Health Center, Budapest, Hungary

*authors contributed equally to the work

Peter Endre Eltes, corresponding author
National Center for Spinal Disorders, Királyhágó St. 1-3, Budapest 1126, Hungary
Tel.:(36) 1-887-7900, Fax.: (36) 1-887-7987, Email address: eltespeter@yahoo.com

CT no.	**DSI** implant construct I_1 vs I_2	**DSI** iliac bone I_1 vs I_2
**1**	0.95	0.96
**2**	0.98	0.96
**3**	0.99	0.96
**4**	1.00	0.97
**5**	1.00	0.96
**6**	0.99	0.97
**7**	0.84	0.96
**8**	0.99	0.96
**9**	0.99	0.96
**10**	0.98	0.97
**11**	0.96	0.96
**12**	1.00	0.97
	mean DSI= 0.97±0.05	mean DSI= 0.96±0.05

Supplementary Table 1. Evaluation of the accuracy of the segmentation process CT (Computed Tomography), DSI (Dice Similarity Index), I (Investigator)

No. of the compared pelvic bone geometry	Hausdorff Distance [mm]			
	min	max	mean	RMS
**1-2**	0.00	3.23	0.50	0.66
**1-3**	0.00	5.30	0.72	0.98
**1-4**	0.00	3.82	0.54	0.70
**1-5**	0.00	3.16	0.57	0.75
**1-6**	0.00	4.33	0.78	0.99
**1-7**	0.00	3.72	0.47	0.64
**1-8**	0.00	5.67	0.49	0.71
**1-9**	0.00	3.65	0.52	0.67
**1-10**	0.00	4.69	0.83	1.06
**1-11**	0.00	4.77	0.80	1.03
**1-12**	0.00	5.01	0.67	0.87
**mean ±SD**	**0.00**	**4.30±0.85**	**0.63±0.14**	**0.82±0.16**

Supplementary Table 2. Evaluation of the accuracy of the alignment process of the pelvic bone, RMS (Root Mean Square), min (minimum), max (maximum)

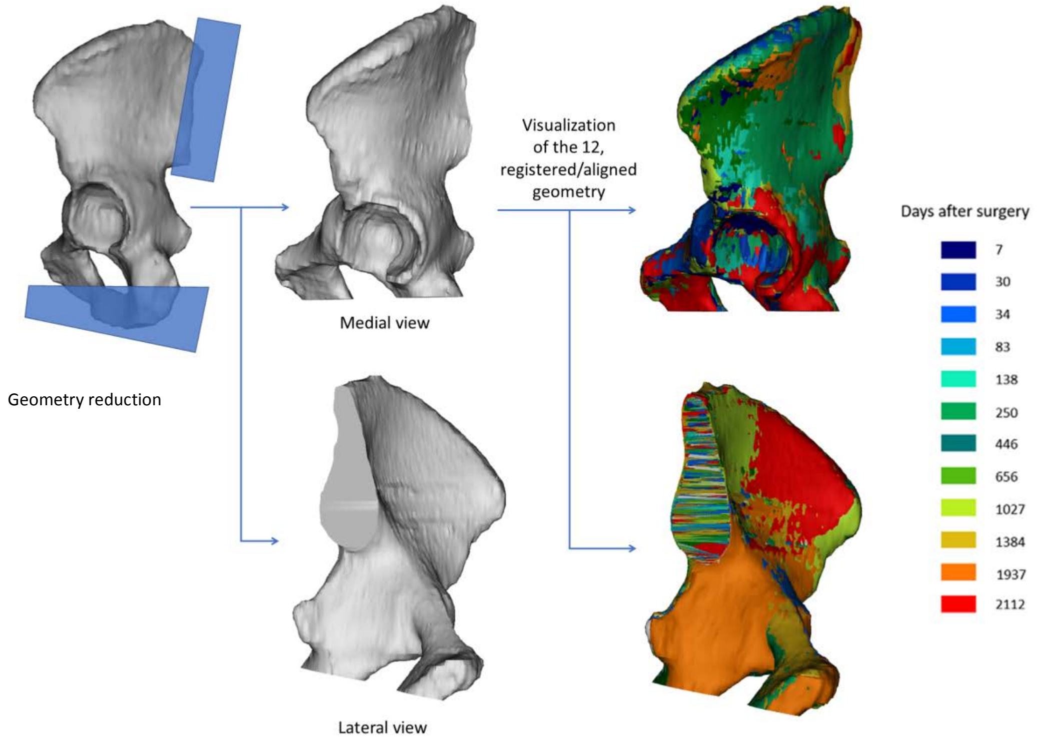

Supplementary Figure 1. Visualization of the 12 reduced, registered/aligned left pelvic bone geometry prepared for Hausdorff Distance measurement. The color code represents the geometry corresponding to the postoperative days on which the CT scan was performed.

No. of the compared iliac screw bodies' geometry	Hausdorff Distance [mm]			
	**min**	**max**	**mean**	**RMS**
**1-2**	0.00	2.28	0.92	1.11
**1-3**	0.00	3.29	0.96	1.27
**1-4**	0.00	2.98	0.95	1.16
**1-5**	0.00	2.90	1.02	1.24
**1-6**	0.00	3.14	1.03	1.24
**1-7**	0.00	4.25	0.90	1.20
**1-8**	0.00	2.94	0.91	1.11
**1-9**	0.00	4.74	0.92	1.26
**1-10**	0.00	3.79	1.18	1.42
**1-11**	0.00	3.21	0.78	1.00
**1-12**	0.00	3.35	0.85	1.09
**mean ±SD**	**0.00**	**3.35±0.68**	**0.95±0.10**	**1.19±0.11**

Supplementary Table 3. Iliac screw bodies' alignment accuracy evaluation. RMS (Root Mean Square), min (minimum), max (maximum), SD (standard deviation)

CT no.	I_1T_1				I_1T_2			
	**X_d** (mm)	**Y_d** (mm)	**Z_d** (mm)	**$3D_d$** (mm)	**X_d** (mm)	**Y_d** (mm)	**Z_d** (mm)	**$3D_d$** (mm)
**1**	87.24	13.89	165.43	187.54	84.58	13.45	165.39	186.25
**2**	92.78	8.99	163.54	188.24	93.73	8.97	161.34	186.81
**3**	91.32	8.80	158.86	183.45	91.02	10.12	158.14	182.74
**4**	99.72	11.39	154.62	184.34	97.62	3.04	153.00	181.52
**5**	96.41	4.63	155.62	183.12	96.57	6.50	154.38	182.21
**6**	96.60	6.20	153.12	181.15	97.10	4.65	156.34	184.10
**7**	89.57	4.90	159.27	182.79	91.33	3.75	157.81	182.37
**8**	93.29	2.38	155.89	181.69	92.81	2.24	157.26	182.62
**9**	89.31	5.83	157.35	181.02	90.15	6.61	157.29	181.41
**10**	95.57	6.01	152.24	179.85	99.93	9.12	152.26	182.35
**11**	93.46	8.14	155.47	181.58	94.21	7.59	155.48	181.95
**12**	96.40	11.72	151.24	179.73	98.76	10.10	150.81	180.55
**mean**	**93.47**	**7.74**	**156.89**	**182.88**	**93.98**	**7.18**	**156.63**	**182.91**
**±SD**	3.65	3.38	4.31	2.72	4.35	3.36	4.00	1.90

Supplementary Table 4. Deformation measurements performed by the first investigator
CT (Computer Tomography), I (Investigator), T (time point), X_d (distance in the coronal plane), Y_d (distance in the axial plane), Z_d (distance in the sagittal plane), $3D_d$ (three-dimensional distance), SD (standard deviation)

CT no.	I_2T_1				I_2T_2			
	**X_d** (mm)	**Y_d** (mm)	**Z_d** (mm)	**$3D_d$** (mm)	**X_d** (mm)	**Y_d** (mm)	**Z_d** (mm)	**$3D_d$** (mm)
**1**	87.64	17.17	165.95	188.45	87.44	16.13	167.12	189.30
**2**	94.33	12.37	160.53	186.60	92.69	8.66	161.93	186.78
**3**	89.70	8.70	159.97	183.61	92.12	10.23	159.65	184.60
**4**	97.19	2.26	154.68	182.69	96.50	3.01	154.60	182.27
**5**	96.50	6.67	155.58	183.20	95.77	5.86	155.66	182.86
**6**	96.35	5.73	154.93	182.54	96.68	5.06	154.06	181.95
**7**	91.43	4.17	158.61	183.12	91.74	3.36	159.10	183.69
**8**	92.75	2.48	157.15	182.50	92.62	2.56	157.11	182.40
**9**	91.05	8.19	157.49	182.10	89.83	4.72	158.27	182.05
**10**	96.44	5.19	154.03	181.80	96.54	4.66	153.69	181.56
**11**	93.43	7.40	154.88	181.03	93.78	7.47	155.25	181.53
**12**	95.55	9.27	151.71	179.53	95.52	9.29	151.68	179.49
**mean**	**93.53**	**7.47**	**157.13**	**183.10**	**93.44**	**6.75**	**157.34**	**183.21**
**±SD**	3.07	4.21	3.78	2.37	2.93	3.88	4.23	2.63

Supplementary Table 5. Deformation measurements performed by the second investigator , CT (Computer Tomography), I (Investigator), T (time point), X_d (distance in the frontal plane), Y_d (distance in the coronal plane), Z_d (distance in the sagittal plane), $3D_d$ (three-dimensional distance)

CT no.	I_3T_1				I_3T_2			
	**X_d** (mm)	**Y_d** (mm)	**Z_d** (mm)	**$3D_d$** (mm)	**X_d** (mm)	**Y_d** (mm)	**Z_d** (mm)	**$3D_d$** (mm)
**1**	87.63	16.26	167.23	189.50	86.87	15.63	167.36	189.21
**2**	93.24	9.86	161.12	186.42	92.89	9.29	160.84	185.97
**3**	92.38	10.25	160.20	185.21	92.24	10.33	158.73	183.88
**4**	97.74	4.84	155.48	183.71	97.19	3.27	154.88	182.88
**5**	96.65	5.85	154.66	182.47	97.18	7.60	156.05	183.99
**6**	96.68	5.54	153.71	181.67	96.00	4.14	154.45	181.90
**7**	91.39	4.34	158.59	183.09	90.47	3.76	160.09	183.92
**8**	92.13	2.81	156.67	181.77	92.68	3.00	157.16	182.48
**9**	88.83	6.77	158.29	181.64	88.62	6.38	159.43	182.52
**10**	96.48	5.66	153.08	181.04	95.64	4.84	153.88	181.24
**11**	93.34	8.05	155.51	181.55	93.05	7.91	155.54	181.42
**12**	97.45	10.53	150.92	179.96	96.94	10.53	152.23	180.78
**mean**	**93.66**	**7.56**	**157.12**	**183.17**	**93.31**	**7.22**	**157.55**	**183.35**
**±SD**	3.39	3.68	4.36	2.69	3.43	3.78	4.08	2.35

Supplementary Table 6. Deformation measurements performed by the third investigator
CT (Computer Tomography), I (Investigator), T (time point), X_d (distance in the coronal plane), Y_d (distance in the axial plane), Z_d (distance in the sagittal plane), $3D_d$ (three-dimensional distance), SD (standard deviation)

Supplementary study 1: Gait evaluation after total sacrectomy

Introduction

During the en-block sacrectomy surgery of the presented patient, the lumbosacral intervertebral disc was resected, and the dural sac (together with the cauda equina) was cut through immediately below the L5 origins. Bilaterally the cranial and ventral ligaments of the SI joints and the nerve roots (bilaterally below the S1) were both cut through at the lateral aspect of the tumour (1). However, the patient was able to walk with crutches at 3 months FU, and without any assisting device at 12 months FU. In order to quantify and evaluate the gait of the patient at the 6 year FU, gait analysis was performed.

Method

Gait data were acquired while the patient walked along a straight path at a self-selected speed, and five walking repetitions were completed per side. The subject was fitted with the VICON plug-in-gait marker setup based on the Davis gait model (2) with markers mounted on the segments of interest at the pelvis, left and right thighs, shanks and feet. Three-dimensional kinematic data were recorded using a 6-camera system (MXT40, VICON, UK). Kinetic data were acquired using one force platform (AMTI OR6, USA) mounted halfway along the path. Lower limb kinematics and kinetics were calculated using NEXUS (VICON, UK) and compared to normative data (3). The acquired results were then plotted to show the mean angle of motion and the standard deviation associated with each variable of the left and right leg of the patient and the normative data.

Result

The patient was able to walk independently with minor gait alterations to compensate the lost neural functions (**Supplementary study Figure 1**). During the FU, no radiological sign of implant failure was registered. The gait was slow and asymmetric with more support on the left side. Joint mobility was close to the normative data in all joints, particularly in the distal joints.. A forward leaning of 20° was seen at the level of the pelvis and trunk throughout the gait cycle. Adduction moments increased at the hip on both sides while joint moments decreased at the knee. Joint power analysis showed a decrease in propulsion power at the hip and ankle.

Discussion/Conclusion

The Closed-Loop reconstruction technique can provide excellent locomotor outcomes after total sacrectomy. A similar result was demonstrated by Smith et al. (4). The fact that the patient was able to walk resulted in a periodic cyclical load of the construct.

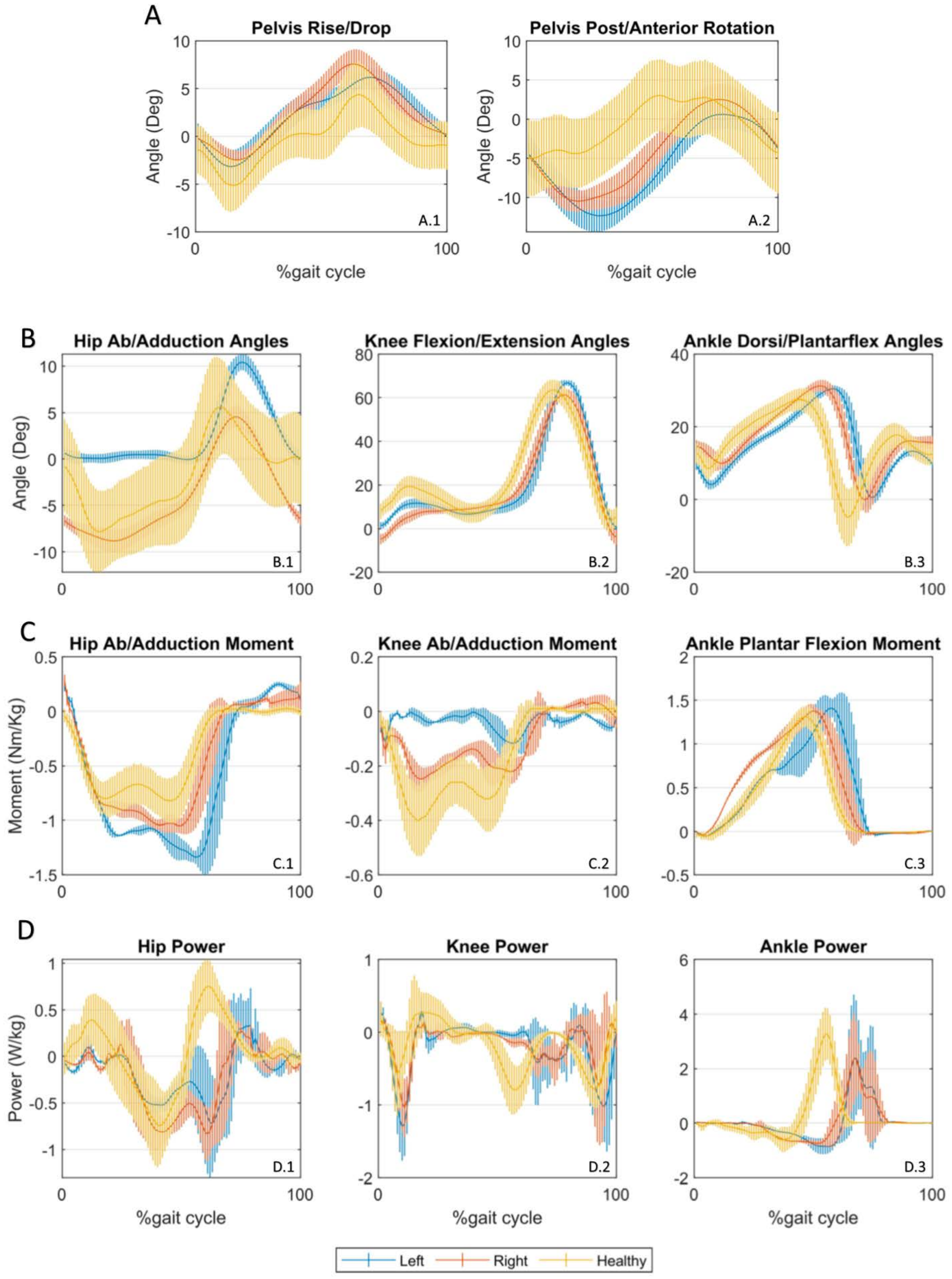

Supplementary study I., Figure 1. Gait evaluation at 5 years after total sacrectomy. Time-series Kinematics (**A**, **B**) and Kinetics (**C, D**) of the pelvis, hip, knee and ankle compared to healthy subjects from the literature (3). The data were normalized to 100% of the gait cycle, kinetic data were normalized to body weight. Vertical lines along the curves indicate the standard deviation. Positive values correspond to pelvic hike (**A.1**), pelvis posterior rotation (**A.2**), hip abduction (**B.1**), knee flexion (**B.2**), ankle dorsiflexion **(B.3)**, hip and knee abductor moments **(C.1,2)** and ankle plantar flexor moment (**C.3**). Plots in (**D**) indicate the joint power in the hip, knee and ankle during flexion/ extension.

Reference:

1. Varga PP, Lazary A. Chordoma of the sacrum: “en bloc” total sacrectomy and lumbopelvic reconstruction. *Eur Spine J* (2010) **19**:1039–1040. doi:10.1007/s00586-010-1460-4

2. Davis III RB, Ounpuu S, Tyburski D, Gage JR. A gait analysis data collection and reduction technique. *Hum Mov Sci* (1991) **10**:575–587.

3. Bovi G, Rabuffetti M, Mazzoleni P, Ferrarin M. A multiple-task gait analysis approach: kinematic, kinetic and EMG reference data for healthy young and adult subjects. *Gait Posture* (2011) **33**:6–13.

4. Smith JA, Tuchman A, Huoh M, Kaiser AM, Schooler WG, Hsieh PC. Locomotor biomechanics after total sacrectomy: a case report. *Spine (Phila Pa 1976)* (2014) **39**:E1481–E1487.

Supplementary study II.: Right pelvic bone, and right trans iliac screws evaluation for the coordinate system selection

Introduction

The aim of this supplementary study was to investigate the use of the right side pelvic bone and iliac screws as the basis of a common coordinate system for the implant contour deformation measurement.

Method

Image processing, 3D geometry definition, alignment of the implant construct geometries

Segmentation process was performed on the CT images (1). For this, the thresholding algorithm and manual segmentation tools (erase, paint, fill etc.) in Mimics® image analysis software (Mimics Research, Mimics Innovation Suite v21.0, Materialise, Leuven, Belgium) were used. (**Supplementary study II., Figure 1. A**). During the segmentation process, the bone volume was first separated from the surrounding soft tissue by thresholding, based on HU (Hounsfield Unit) levels. The left pelvic bone was isolated, and then the implant geometry was separated as well. The resulting masks (a group of voxels) were homogeneously filled, preserving the outer contour of the geometrical border in 2D. From the masks, a triangulated surface mesh was automatically generated for the iliac bone and for the implant construct (**Supplementary study II., Figure 1. B**).

To determine the implant construct's rod displacement and rotation, the 12 segmented implant geometry with the right pelvic bone were aligned in the same coordinate system. The first postop CT scan based right pelvic bone was used as reference geometry. A control points based rigid registration algorithm was used via Mimics® software. The 8 control points corresponded to easily identifiable anatomical landmarks at the right pelvic bone (**Supplementary study II., Figure 1. B)**. During the registration, the implant construct moved together with the iliac bone (**Supplementary study II., Figure 1. C)**. The trans-iliac screw bodies' geometry overlapped, after the registration of the iliac bones. The axes of the iliac screws were collinear and coincident (**Supplementary study II., Figure 1. E**, **F**).

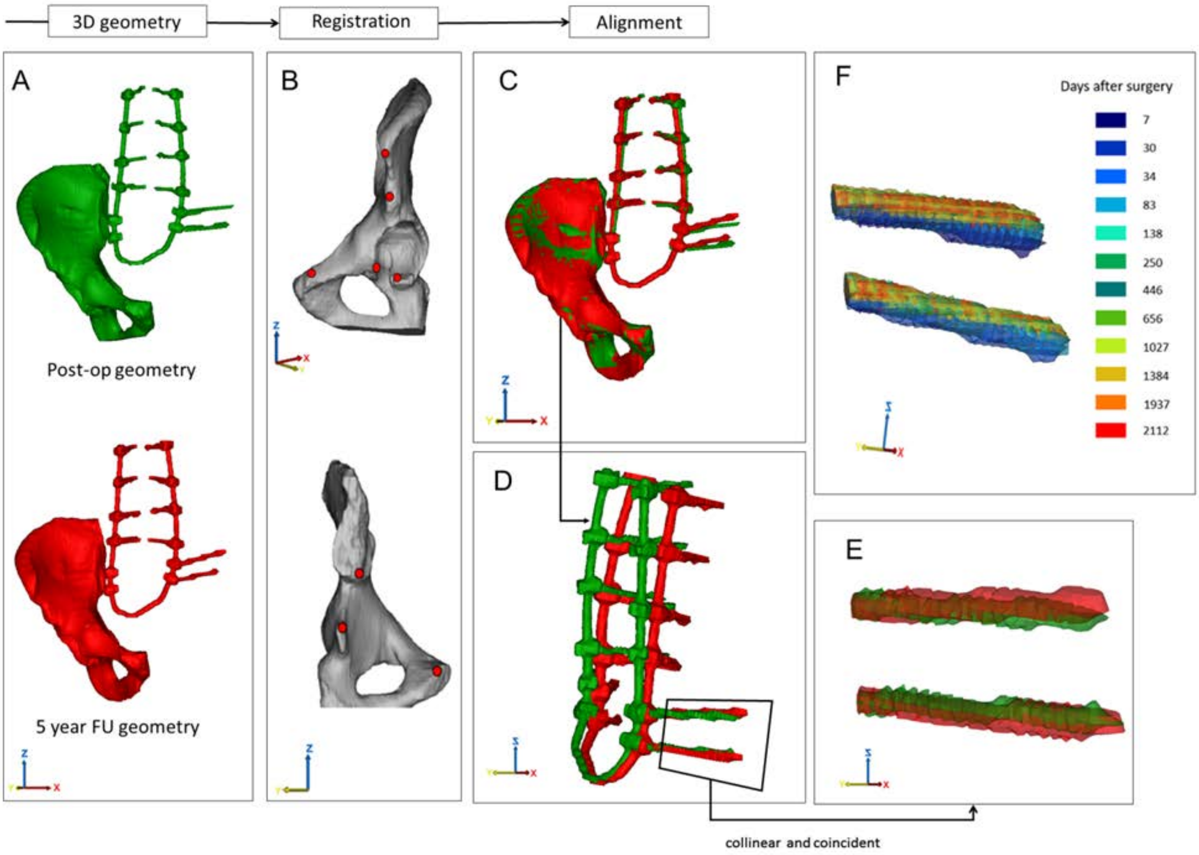

Supplementary study II., Figure 1: Postop CT scan-based geometry definition and alignment. **A** thresholding based segmentation was performed on the postop CT scan in order to define the right pelvic bone and the implant construct. **B** 8 points corresponding to anatomical landmarks were used for the simultaneous registration of the iliac bone and implant construct geometry. **C** every postop right pelvic bone + implant construct geometry was registered to the first postop geometry. **E** the trans-iliac screw bodies' geometry overlapped after the pelvic bone registration. The axes of the iliac screws were considered to be collinear and coincident. **F** geometric overlap of the bodies of the iliac screws after the alignment process. The 12 postop CT scan-based surface meshes representing the iliac screw bodies are color-coded corresponding to the scale bar (color= CT scan session and number of days after surgery). The surfaces mesh is visualised with 75% transparency.

Implant deformity measurements

The implant construct's geometry was considered as a tubular structure and the centreline of the geometry was defined with the Mimics® Software (**Supplementary study II., Figure 2. A**). A "mobile" point corresponding to the L2 right pedicle screw tip and a fixed point was selected in the centreline corresponding to the tip of the caudal right iliac screw. The distances between the points were measured in three anatomical planes (**Supplementary study II., Figure 2. B, C, D**) using 3-matic® software (Mimics Innovation Suite v21.0, Materialise, Leuven, Belgium).

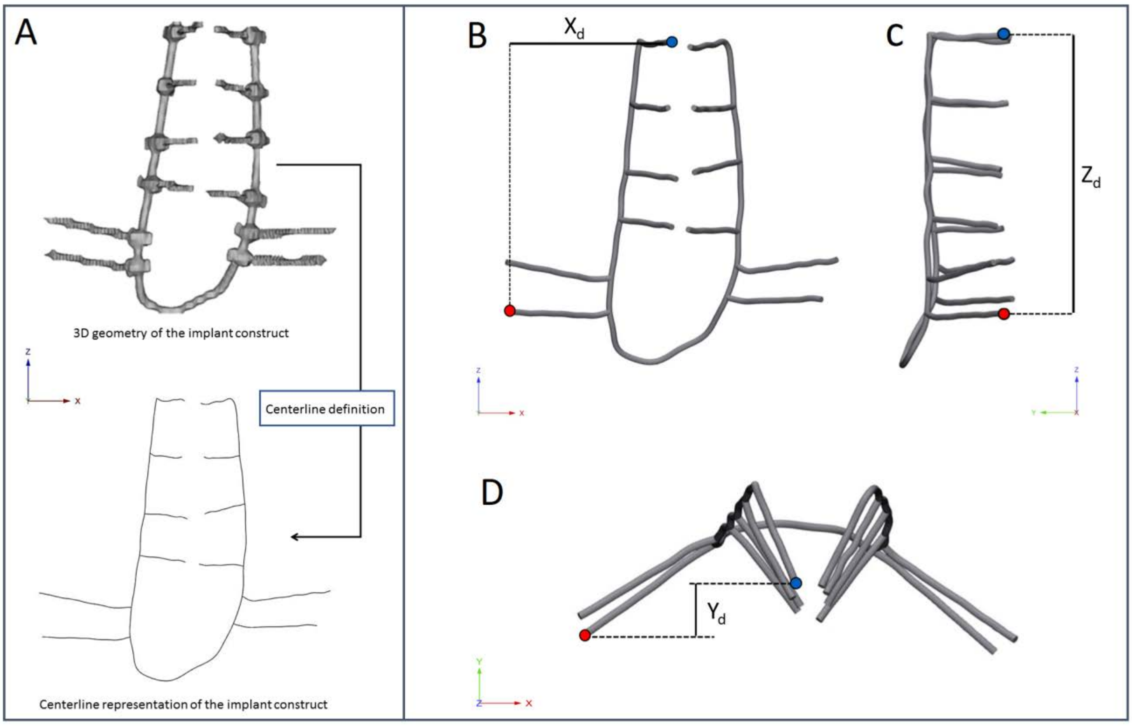

Supplementary study II., Figure 2: Implant construct's geometry simplification and contour deformation measurement. **A** the segmented geometry of the implant construct was considered a tubular structure, the centreline of the geometry was defined. **B, C, D** a fixed point (red dot) was selected in the centreline corresponding to the tip of the caudal right side trans iliac screw, and a mobile point (blue dot) corresponding to the L2 right pedicle screw tip. The distance between the points was determined **B** in the coronal plane (X_d), **C** sagittal plane (Z_d), **D** axial plane (Y_d).

Statistical analysis

All statistical tests were performed with SPSS statistical package version 23 (SPSS Inc, Chicago, IL). The relationships between the implant deformation in the anatomical planes and the number of postop days were analysed by the Spearman's rank correlation (Supplementary study II., Figure 4.).

Result

After the iliac bone alignment, in order to demonstrate the colinear and coincident position of the iliac screw axes, the geometric overlap of the body of the iliac screws was visualised in **Supplementary study II., Figure 3**.

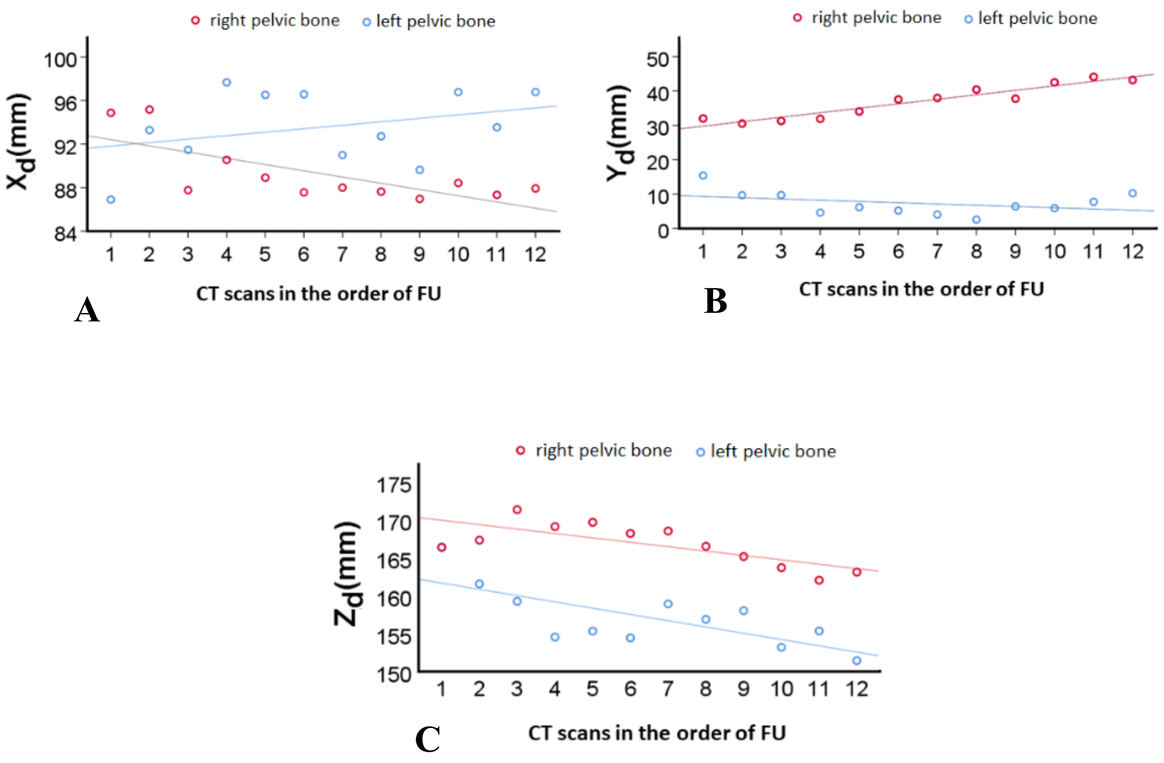

Supplementary study II., Figure 3: Implant construct contour deformation measurement on the 12 post op CT scans with right (red circle) and left pelvic bone (blue circle) used as the basis of the coordinate system. **A** in the coronal plane (X_d) we can see a right bending tendency of the construct, followed by the trend lines. **B** axial plane (Y_d) measurements, the trend lines shows symmetry, with moderate upward slope in the case of right pelvic bone registration. **C** sagittal plane (Z_d) measurements, symmetric downward slope orientation of the trend lines.

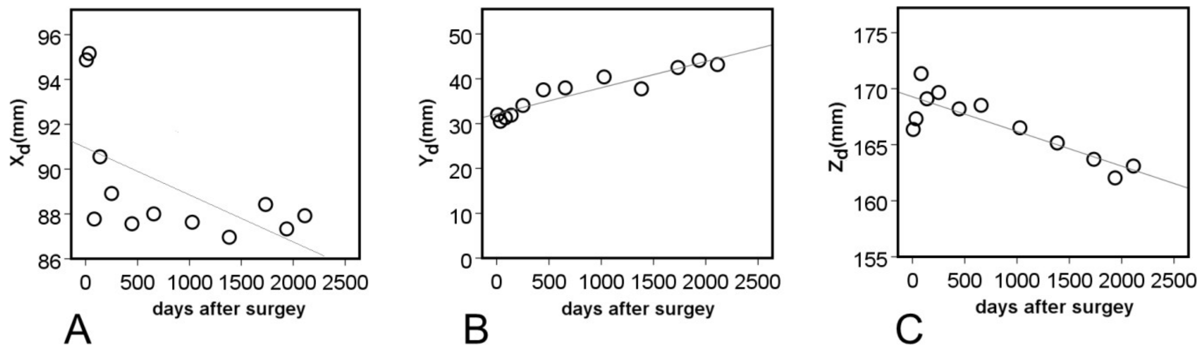

Supplementary study II., Figure 4. Association between the distance of the mobile (L2 right pedicle screw tip) point from the fixed point (right, caudal trans iliac screw tip) in the anatomical planes, and the number of days after surgery (DAS). **A** significant negative, strong correlation was found between the X_d (frontal plane) and DAS (ρ=-0.629, p=0.028). **B** significant, negative, very strong correlation was found between the Y_d (axial plane) and DAS (ρ=0.93, p<0.0001). **C** significant, negative, strong correlation was found between the Z_d (sagittal plane) and the DAS (ρ=-0.678, p=0.015).

The implant deformation was defined by measuring the distance in the three anatomical planes between the right L2 pedicle screw tip and the right caudal iliac screw tip. The measurements are presented on **Supplementary study II., Figure 4.**

The mean change in the dimensions compared to the first postop CT scan was ΔX_d=6.18±2.18 mm for the frontal plane, ΔY_d=5.80±4.35 mm for the coronal plane, ΔZ_d= 2.51±1.45 mm for the sagittal plane.

The association between the average $X_d/Y_d/Z_d$ measurement and the number of days after surgery is shown in **Supplementary study II., Figure 4,** the interpretation of the correlation was based Evans et al. 1996 work (2). Significant, negative, strong correlation was found between the X_d (frontal plane) contour deformation and the days after surgery (ρ=-0.629, p=0.028), significant, pozitive, very strong correlation was found between the Y_d (axial plane) and the days after surgery (ρ=0.93, $p<0.0001$), significant, negative, strong correlation was found between the Z_d (sagittal plane) and the number of days after surgery (ρ=-0.678, p=0.015).

Discussion/Conclusion

The findings of this supplementary study demonstrate that the right side iliac screw body's does not deform or change its position compared to the firs post-op surgery CT scan, during the FU similarly to the left side (main study, manuscript). Theoretically, any point in these two screw body geometries can be used as a reference point in a measurement process. The Z_d contour deformation demonstrates the forward bending tendency of the construct over the FU, similarly to the main study in the manuscript. However the X_d and Y_d contour deformation is different, compared to the left pelvic bone registrations, with significant strong and very strong correlations with the number of days after surgery.

Reference:

1. Bozic KJ, Keyak JH, Skinner HB, Bueff HU, Bradford DS. Three-dimensional finite element modeling of a cervical vertebra: an investigation of burst fracture mechanism. *J Spinal Disord* (1994) **7**:102–110.

2. Evans JD. *Straightforward statistics for the behavioral sciences.* Thomson Brooks/Cole Publishing Co (1996).
